# Effect of cigarette smoking on the washout time of sevoflurane anesthesia

**DOI:** 10.1186/1471-2253-10-8

**Published:** 2010-06-22

**Authors:** Tayfun Adanir, Aynur Atay, Atilla Sencan, Murat Aksun, Nagihan Karahan

**Affiliations:** 1 Ataturk Training and Research Hospital, 2nd Department of Anesthesiology and Reanimation, Izmir, Turkey

## Abstract

**Background:**

Cigarette smoking affects the pharmacodynamic and pharmacokinetic behavior of many drugs and causes deterioration of pulmonary mechanics. We have evaluated the effect of cigarette smoking on washout time after one minimum alveolar concentration-h (1 MAC-h) of sevoflurane anesthesia.

**Methods:**

We investigated the washout time of sevoflurane in 30 non-smoking and 30 healthy cigarette smoking (≥20 cigarettes/day for>1 year) ASA I-II physical status patients, aged 18-63 years, who were candidates for otorhinolaryngologic elective surgery under 1MAC-h standardized sevoflurane anesthesia. At the end of the surgery, the sevoflurane vaporizer was turned off and the time taken for the sevoflurane concentration to decrease to MAC-awake (0.3) and 0.1 MAC levels were recorded. In addition, the ratio of the fractions of inspired concentration (Fi) and expired concentration of sevoflurane (Fexp) at 1 MAC and Fexp of sevoflurane at 0.1MAC were recorded. The patients were mechanically ventilated during the washout time.

**Results:**

We found no difference between the 2 study groups with regard to washout time of sevoflurane. The times of 1MAC down to MAC-awake (106 ± 48 sec in non-smokers vs 97 ± 37 sec in smokers, *p *> 0.05) and down to 0.1MAC (491 ± 187 sec in non-smokers vs 409 ± 130 sec in smokers, *p *> 0.05) were similar. Similarly, there were no significant differences in the ratios of Fi/Fexp at 1MAC (1.18 in non-smokers vs. 1.19 in smokers, *p *> 0.05) and Fexp of sevoflurane at 0.1MAC (0.26 in non-smokers vs. 0.25 in smokers, *p *> 0.05).

**Conclusions:**

Washout time of 1MAC-h sevoflurane anesthesia is not appear to be effected by cigarette smoking in patients without significant pulmonary disease.

## Background

Cigarette smoking is a priority concern in perioperative management [1-3] because it may promote local lung inflammation that leads to lung dysfunction. Inhalation of cigarette smoke promotes pulmonary inflammation by inducing chemotaxis, retention, and activation of neutrophils and macrophages [4,5]. The inflammatory process is probably essential for the development of lung injury and in the ensuing impairment of lung function [6,7]. However, the pathologic response to cigarette smoke varies among individuals [8].

Chronic exposure to cigarette smoke affects the metabolism of several drugs, including those anesthetics used as muscle relaxants. Cigarette smoking may also affect the sensitivity of the central nervous system to psychoactive drugs, e.g. benzodiazepines and anesthetics [9,10].

Cigarette smoke contains over 4800 pharmacologically active substances with chronic exposure producing multiple physiological effects. Cigarette smoking also affects the pharmacodynamic and pharmacokinetic behavior of many drugs. In addition, 5% inhaled sevoflurane in humans is metabolized, predominantly by the cytochrome P450 pathway (CYP2E1) induced by cigarette smoking [11].

For these reason, the washout time of sevoflurane may be disturbed in smokers. We have evaluated the effects of cigarette smoking on the washout time after one minimum alveolar concentration-h (1 MAC-h) sevoflurane anesthesia.

## Methods

### Patients

Having obtained the approval by the Local Ethics Committee and written informed consent, the study was conducted by researchers among 60 American Society of Anesthesiologists (ASA) physical status I or II patients scheduled for otorhinolaryngologic surgery. We investigated the washout time of sevoflurane in 30 life-long non-smokers (never having smoked in their lives) and 30 cigarette smokers (≥20 cigarettes/day for>1 year) patients. We assessed the two groups by via questioning them and their relatives (wife, husband, mother, dad or children). The patients were 18-63 years old, with body mass indices ranging from 22 to 27. They were candidates for otorhinolaryngologic elective surgery under 1MAC-h standardized sevoflurane anesthesia. Patients with preexisting pulmonary failure, cardiac failure, hepatic failure, renal failure or pulmonary hypertension and more 1 h-long otorhinolayngologic elective surgical procedures were excluded. All cigarette smokers were assessed for pulmonary failure based on clinical assessment, blood gas pressures, chest x-ray and pulmonary function tests.

### Study design

Patients were not allowed to smoke the day of surgery. Neither group received pre-medication. After being taken to the operation room, all the patients were monitored continuously throughout the study by standard ASA monitors. In addition to standard ASA monitors, inspired oxygen concentration (FiO_2_), inspired and expired concentration of sevoflurane, and MAC levels of the patients were monitored. An 18-gauge cannula was inserted into the antecubital vein to infuse 0.9% NaCl during surgery.

Anesthesia was induced by pentothal (5-7 mg.kg^-1^) and fentanyl (1,5-2 μgr.kg^-1^). The patients' tracheas were intubated by the application of rocuronium (0,6 mg.kg^-1^). General anesthesia was maintained by remifentanyl infusion (0,25-1 μg.kg^-1^.min^-1^) and sevoflurane. Fresh gas flow was 4 lt.min^-1^. During general anesthesia, the end tidal concentration of sevoflurane was maintained at 1 MAC (corrected for the patients' age). The duration of 1 MAC anesthesia of the operations was maintained for 1-h. Patients were mechanically ventilated (V_T_: 6-8 ml. kg^-1^, f: 12.min^-1^) adjusted to provide an end-tidal CO_2 _concentration of 35-40 mmHg and SpO_2 _> 95% with 50% N_2_O in oxygen, during the surgical procedures. The patients' non-invasive arterial tension and heart rate were maintained within normal limits. All data were recorded on each patient's observation chart.

At the end of the surgery, the sevoflurane vaporizer was turned off and the time taken for the sevoflurane concentration to decrease from 1MAC to 0.3 MAC (MAC-awake) and 0.1 MAC were recorded. In addition, the ratio of the fraction of inspired concentration (Fi) of sevoflurane and of expired concentration (Fexp) of sevoflurane at 1 MAC and Fexp of sevoflurane at 0.1MAC were recorded. We measured the ratio of Fi/Fexp of sevoflurane at 1MAC just before the sevoflurane vaporizer was turned off. Because alterations of alveolar ventilation and fresh gas flow would affect both the expired and inspired sevoflurane concentrations at that time; the fresh gas flows, tidal volumes and breathing frequency of the patients were maintained at 6 lt. min^-1^, 6 ml. ideal kg^-1 ^and 12 breath.min^-1^, respectively. Mechanical ventilations of the patients were maintained until extubation and standardized according to ideal body weight. All patients were extubated after the last measurement.

Because it was considered unethical not to use any agent in intubated patients at MAC-awake or 0.1 MAC, we used remifentanil untill the patients' MAC values were decreased to 0.1 MAC. Remifentanil infusion was stopped when the 0.1 MAC level was reached.

### Statistics

Statistical analysis was made by using the SPSS 15.0 (SPSS Inc., Chicago, IL) statistical program. Data were expressed as mean ± SD. Statistical analysis utilized an independent sample *t*-test for all variables. *P *< 0.05 was considered significant. We estimated that a group of at least 30 patients was adequate for parametric independent sample *t*-test.

## Results

**Figure 1:**
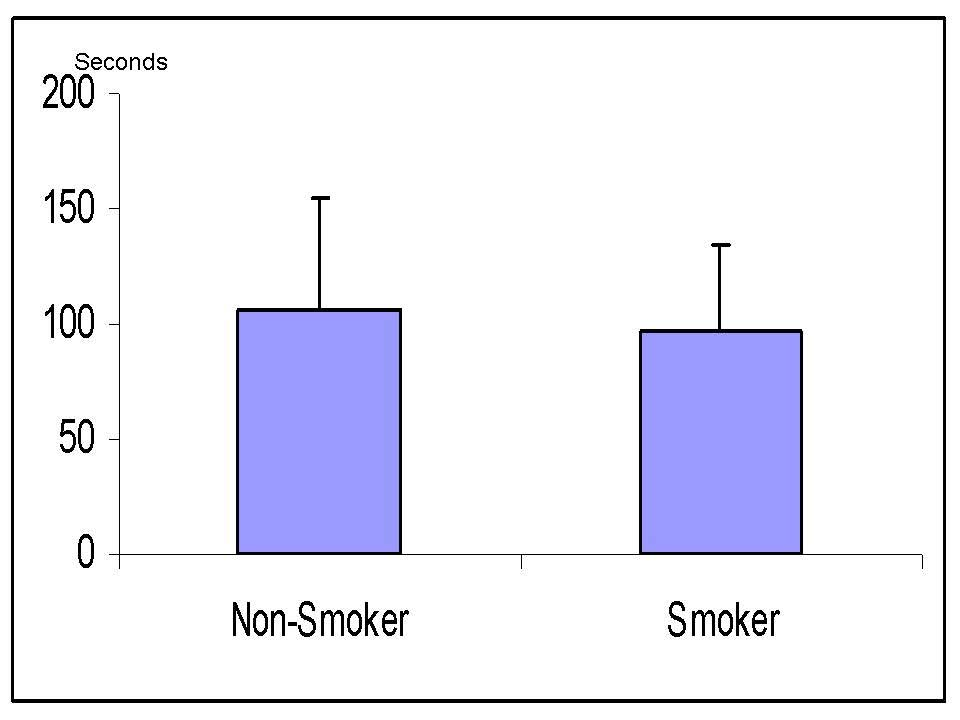
**The time taken for sevoflurane concentration to decrease to 0.3 MAC (MAC-awake) (seconds)**.

**Figure 2:**
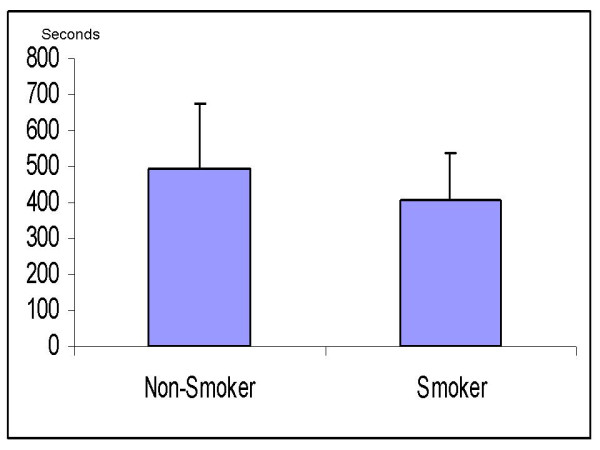
**The time taken for sevoflurane concentration to decrease to 0.1 MAC (seconds)**.

**Figure 3:**
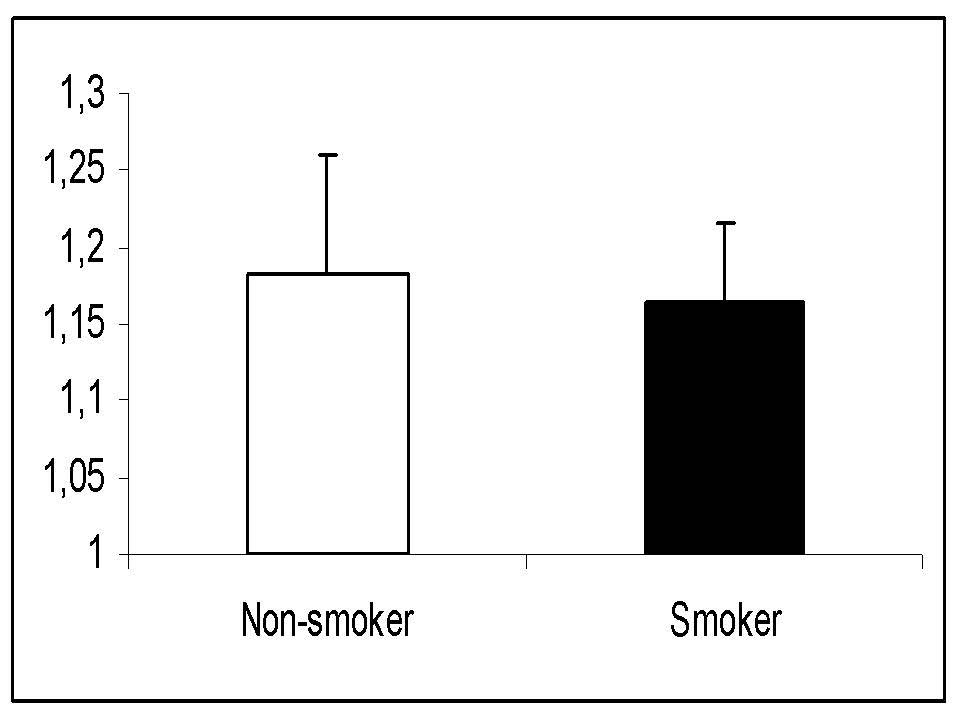
**The ratio between inspired (Fi) and expired sevoflurane concentrations (Fexp) during surgery (Fexp/Fi) at 1 MAC**.

**Figure 4:**
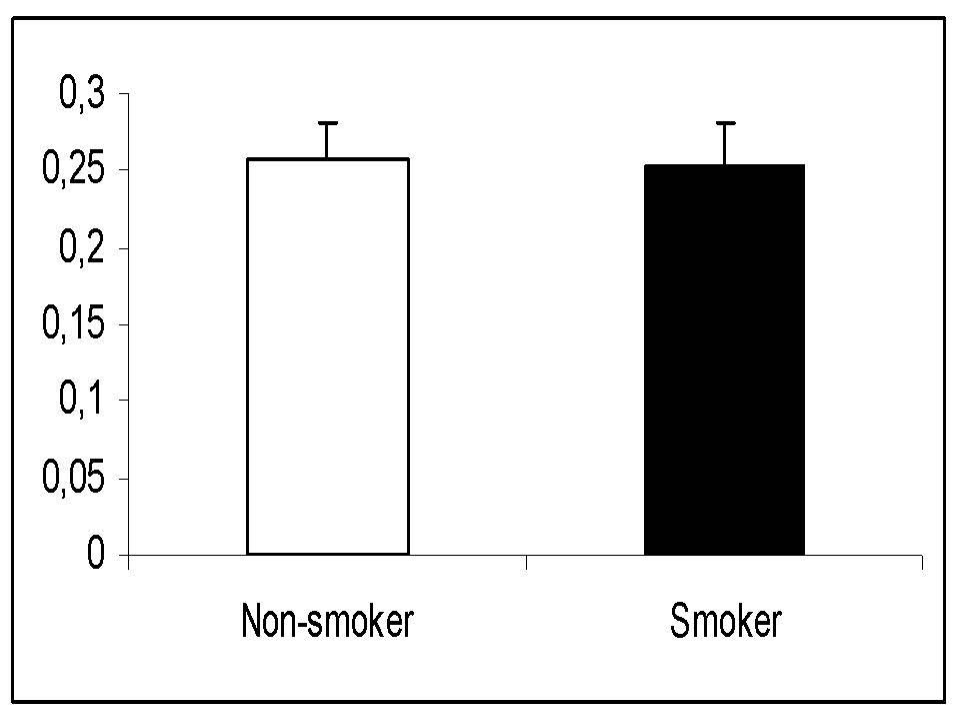
**The fraction of expired sevoflurane concentration (Fexp) at 0.1 MAC**.

Body mass index, age, weight and sex were similar in the smoker and non-smoker groups (Table 1). We found no significant differences between the 2 study groups regarding the washout time of sevoflurane. The times from 1MAC down to MAC-awake (106 ± 48 sec in non-smokers vs. 97 ± 37 sec in smokers; p = 0.67) and 0.1MAC (491 ± 187 sec in non-smokers vs. 409 ± 130 sec in smokers, p = 0.08) were similar (Figs. 1 and 2). In addition, there was no significant difference in the ratios of Fi/Fexp at 1MAC between non-smokers and smokers (1.18 vs. 1.19 at 1MAC respectively, p = 0.23) (Fig. 3). Fexp of sevoflurane at 0.1MAC of the groups were also similar (0.26 in non-smokers vs. 0.25 in smokers, p = 0.75) (Fig. 4).

**Table 1:** Characteristics of the patients.

	Smoker	Non-smoker	*p*
**BMI**	25.62 ± 2.2	26.57 ± 3.4	0.34

**Age (years)**	40 ± 10	42 ± 10	0.43

**Weight**	74.3 ± 11.23	77.07 ± 7.65	0.34

**Sex**	18 F, 12 M	17 F, 13 M	0.42

Body mass index (BMI), age, weight and gender of the groups are presented.

Statistical significant is presented as *p *value. F: female, M: male.

## Discussion

Mendonca et al. [12] found that inhalational induction with sevoflurane was safe as an induction technique in 60 cigarette smokers. Similar to findings in other patient groups, the use of a high initial concentration reduced induction time. As inhalational induction time with sevoflurane is not affected by cigarette smoking, washout time of 1MAC-h sevoflurane anesthesia also seems to be uneffected by cigarette smoking in patients without significant pulmonary disease.

Chronic exposure to cigarette smoke affects the metabolism of a number of drugs, including those used in anesthesia, e.g. muscle relaxants [13,14]. The biotransformation of commonly used volatile agents is primarily mediated by CYP2E1 and cigarette smoking also induces this enzyme [11]. The CYP2E family contains only one enzyme, CYP2E1 (previously known as dimethylnitrosamine N-demethylase) which is responsible for the metabolism of small organic compounds, such as alcohol and carbon tetrachloride as well as the halogenated anesthetic agents halothane, enflurane, diethyl ether, trichloroethylene, chloroform, isoflurane and methoxyflurane [15,16]. Since sevoflurane is metabolized by this pathway induced by cigarette smoke, there is the possibility of a postoperative increase in serum inorganic fluoride becomes exaggerated in smokers after the administration of sevoflurane anesthesia. This was not observed in a recent study, so there is no basis for clinical concern [17].

Cigarette smoking may also affect the sensitivity of the central nervous system to psychoactive drugs, such as benzodiazepines and anesthetics. Lysakowski et al. [18] found that higher doses of propofol were required to abolish consciousness compared with non-smokers, supporting the concept that cigarette smoking can alter central nervous system sensitivity. These differences were small, however, and unlikely to be of clinical significance.

Recovery from inhalational anesthesia depends on lowering the concentration of anesthetic in brain tissue. Inhaled anesthetics are eliminated by biotransformation, transcutaneous loss, and exhalation. Hepatic metabolism of sevoflurane contributes approximately 5%, even when cigarette smoking increases many-fold the function of the P450 pathway, which is virtually considered quite low to affect washout kinetics of this volatile agent. The most important route for elimination of this inhalational anesthetic is through lung alveoli. Many of the factors that speed induction also hasten washout time: elimination of rebreathing, high fresh gas flow, low anesthetic-circuit volume, low absorption by the anesthetic circuit, decreased solubility, high cerebral blood flow, and increased ventilation. The speed of washout time also depends on the length of time the anesthetic has been administered [19]. The alveolar ventilation, fresh gas flow and length of time patients were anesthetized were kept constant in our study. However, we found no statistically significant differences between the groups for washout time of sevoflurane. Although there seems to be a tendency toward shorter washout times for the cigarette smokers compared to the non-smokers, clinical significance was not reached between the groups.

Active cigarette smoking also affects lung function indices other than FEV1. In cigarette smokers, neutrophils are typically present in the lower airways. Cigarette smoking is a known risk factor for acceleratingthe decline of lung function in adults. Cigarette smoking has also been associated with several diffuse lung diseases in which both bronchiolar and interstitial lung inflammation seem to result from chronic cigarette smoke inhalation [20]. An association between decrease in diffusion capacity of the lung and cigarette consumption has been observed, even in healthy subjects. Airway hyper-responsiveness also develops in cigarette smokers. We assumed that washout time of inhaled anesthetics is affected through all above results and can effect alveolar ventilation.

Cigarette smoking increases hemoglobin, hemotocrit, plasma fibrinogen, blood pressure and heart rate. It seems to increase blood flow and the elimination of sevoflurane due to the induction of CYP2E1. On the other hand, it seems to decrease the elimination of sevoflurane via the lungs due to affect alveolar ventilation. However, we did not observe any difference of washout time of sevoflurane between the smokers and non-smokers in our study.

Our results might have several limitations that deserve comment. The study was conducted on a moderate sample size and our results may not apply to smokers with lung disease. If we had obtained plasma levels of volatile anesthetics, more accurate data might have been provided. We found no data evaluating washout time of other volatile anesthetics in cigarette smokers. Further studies are needed to evaluate washout time for more and less soluble volatile agents in cigarette smokers.

## Conclusions

Washout time of 1MAC-h sevoflurane anesthesia is not effected by cigarette smoking in patients without significant pulmonary disease.

## Competing interests

The authors declare that they have no competing interests.

## Authors' contributions

TA participated in the design of the study, in the sequence alignment and drafted the manuscript, in the collection and analyzed data. AA participated in the sequence alignment and drafted the manuscript, in the collection and analyzed data. AS participated in the collection and analyzed data. MA participated in the collection and analyzed data. NK participated in analyzed data. All authors have read and approved the manuscript.

## Pre-publication history

The pre-publication history for this paper can be accessed here:

http://www.biomedcentral.com/1471-2253/10/8/prepub
